# Charge neutralization and β-elimination cleavage mechanism of family 42 L-rhamnose-α-1,4-D-glucuronate lyase revealed using neutron crystallography

**DOI:** 10.1016/j.jbc.2024.105774

**Published:** 2024-02-19

**Authors:** Naomine Yano, Tatsuya Kondo, Katsuhiro Kusaka, Takatoshi Arakawa, Tatsuji Sakamoto, Shinya Fushinobu

**Affiliations:** 1 Structural Biology Division, Japan Synchrotron Radiation Research Institute, Hyogo, Japan; 2 Department of Applied Biological Chemistry, Graduate School of Agriculture, Osaka Metropolitan University, Sakai, Osaka, Japan; 3 Neutron Industrial Application Promotion Center, Comprehensive Research Organization for Science and Society, Tokai, Ibaraki, Japan; 4 Faculty of Pharmaceutical Sciences, Tokyo University of Science, Noda, Chiba, Japan; 5 Department of Biotechnology, The University of Tokyo, Bunkyo-ku, Tokyo, Japan; 6 Collaborative Research Institute for Innovative Microbiology, The University of Tokyo, Bunkyo-ku, Tokyo, Japan

**Keywords:** neutron crystallography, polysaccharide lyase, gum arabic, carbohydrate-active enzyme, arabinogalactan protein, X-ray crystallography, carbohydrate processing, polysaccharide

## Abstract

Gum arabic (GA) is widely used as an emulsion stabilizer and edible coating and consists of a complex carbohydrate moiety with a rhamnosyl-glucuronate group capping the non-reducing ends. Enzymes that can specifically cleave the glycosidic chains of GA and modify their properties are valuable for structural analysis and industrial application. Cryogenic X-ray crystal structure of GA-specific L-rhamnose-α-1,4-D-glucuronate lyase from *Fusarium oxysporum* (FoRham1), belonging to the polysaccharide lyase (PL) family 42, has been previously reported. To determine the specific reaction mechanism based on its hydrogen-containing enzyme structure, we performed joint X-ray/neutron crystallography of FoRham1. Large crystals were grown in the presence of L-rhamnose (a reaction product), and neutron and X-ray diffraction datasets were collected at room temperature at 1.80 and 1.25 Å resolutions, respectively. The active site contained L-rhamnose and acetate, the latter being a partial analog of glucuronate. Incomplete H/D exchange between Arg166 and acetate suggested that a strong salt-bridge interaction was maintained. Doubly deuterated His105 and deuterated Tyr150 supported the interaction between Arg166 and the acetate. The unique hydrogen-rich environment functions as a charge neutralizer for glucuronate and stabilizes the oxyanion intermediate. The NE2 atom of His85 was deprotonated and formed a hydrogen bond with the deuterated O1 hydroxy of L-rhamnose, indicating the function of His85 as the base/acid catalyst for bond cleavage *via* β-elimination. Asp83 functions as a pivot between the two catalytic histidine residues by bridging them. This His–His–Asp structural motif is conserved in the PL 24, 25, and 42 families.

Arabinogalactan proteins (AGPs) are a family of proteoglycans in the plasma membrane, extracellular matrix, and cell walls of various plants (1, 2). AGPs play essential roles in many plant physiological processes, including cell death, cell elongation, stress response, intercellular adhesion, and intercellular signal transduction (3). AGPs comprise over 90% type II arabinogalactan (a carbohydrate moiety) and a core protein rich in hydroxyproline. Type II arabinogalactan contains β-1,3-galactan as the main chain and β-1,6-galactooligosaccharide side chains, modified with various sugars, including L-rhamnose (Rha) and D-glucuronic acid (GlcA) (1, 4, 5, 6). The complicated polysaccharide structure of AGPs with many branches hampers the detailed analysis of their structure-function relationships (7). Gum arabic (GA) is a subclass of type II arabinogalactan that is sometimes regarded as a representative AGP (6). GA is a sticky exudate from *Acacia* trees produced under stress, such as drought and mechanical injury (8). Therefore, GA is widely used as an emulsion stabilizer and coating in various applications, such as food, drink, ink, and pharmaceutical industries (9, 10, 11). The primary structure of GA consists of disk-like star-shaped nanoparticles (12). The polysaccharide structure of GA has been analyzed using chemical methods and NMR analysis (13, 14); however, its detailed structure has not yet been elucidated. The non-reducing ends of the GA side chains are often capped with α-L-rhamnose-(1→4)-D-glucuronic acid (Rha-GlcA); therefore, enzymes targeting the disaccharide cap structure would be promising tools for elucidating the detailed structure and function of GA and modifying its physical properties.

We have studied various GA-degrading enzymes from *Fusarium oxysporum* 12S, a phytopathogenic fungus that can grow using GA as its sole carbon source (15, 16, 17, 18, 19). L-Rhamnose-α-1,4-D-glucuronate lyase (EC 4.2.2.28) from *F. oxysporum* 12S (FoRham1) cleaves the Rha-GlcA glycosidic bond present in the GA side chain by β-elimination to generate Δ4,5-unsaturated GlcA at the non-reducing end (20). We previously reported the X-ray crystal structure of FoRham1 (20). The structures of wild-type (WT) enzymes in ligand-free and Rha complex forms were determined at 1.05 and 1.40 Å resolutions, respectively. The structure of an inactive H105F mutant in complex with Rha-GlcA was determined at 2.42 Å resolution (20). We identified the active site of FoRham1 on the anterior side of a seven-bladed β-propeller domain and found structural similarities to several polysaccharide lyase (PL) families, including the catalytic residues. Therefore, FoRham1 and its homologs in the former glycoside hydrolase 145 family were reclassified into a novel PL42 family in the Carbohydrate-Active enZYmes database (http://www.cazy.org) (21). Based on structural and mutational analyses, we proposed a possible catalytic mechanism for FoRham1 that involves charge neutralization of uronate carboxylate by Arg166 and proton abstraction and donation by His85 in the lytic cleavage of the O4–C4 bond (20). His105 may support the base-acid dual function of His85. However, this reaction mechanism was proposed for X-ray structures that do not contain hydrogen (H) atoms, although base/acid catalysis should be involved in the enzymatic reaction. In X-ray crystallography, the visibility of atoms depends on their atomic numbers because X-rays interact with electrons in the protein and are scattered. As each H atom contains only one electron, the signals obtained from hydrogen atoms are weaker than C, N, O, and S atoms. This makes the determination of hydrogen atom positions in proteins challenging except for ultra-high resolution. Neutron crystallography is a powerful method for determining the H and deuterium (D) atom positions in proteins (22). However, it requires prolonged beam exposure and a larger volume of crystals (>1 mm^3^) because the neutron beam intensity is substantially weaker than that of X-rays. Neutrons interact with atomic nuclei and are not scattered depending on atomic numbers. The neutron scattering lengths of H and D atoms are comparable to those of C, N, O, and S atoms. In this study, we performed joint X-ray/neutron (XN) crystallography of FoRham1 to propose a detailed reaction mechanism based on protonation around the active site. Many crystal structures of carbohydrate-active enzymes have been determined using X-ray (21). In particular, carbohydrate-active enzymes generally operate with general acid/base catalysis that involves the movement of protons, locating hydrogen atoms in their three-dimensional structure is highly relevant for understanding the catalytic mechanism. However, to the best of our knowledge, this is the first report on the neutron structure of a PL, of which there are over 40 families.

## Results and discussion

### Diffraction experiments and joint XN structure determination

**Figure 1:**
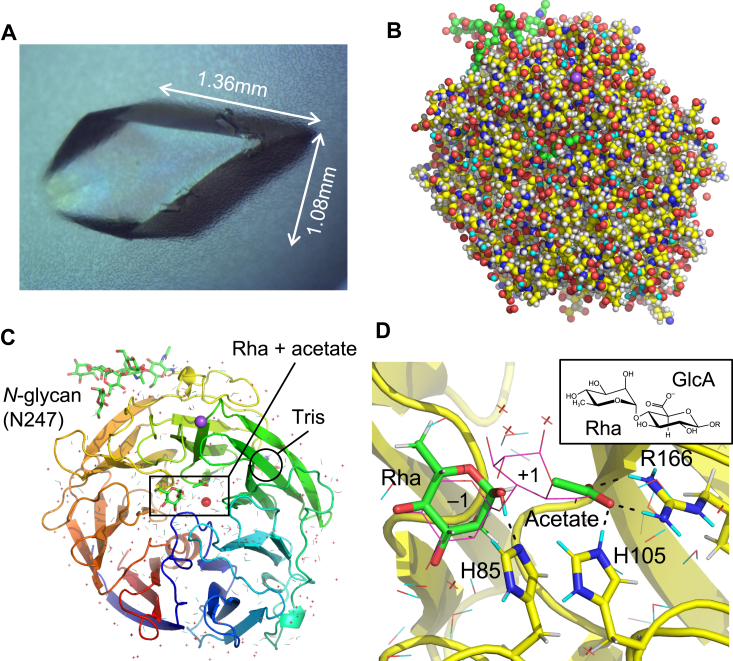
**Joint XN structure of FoRham1.***A*, huge crystal used in crystallography. *B*, the overall structure is illustrated by *sphere* representation. Atoms are colored as; C (*yellow* for protein and *green* for ligands and *N*-glycan), O (*red*), N (*blue*), Na^+^ (*purple*), H (*white*), and D (*cyan*). *C*, the overall structure is demonstrated by a rainbow-colored ribbon viewed from the anterior side of the β-propeller domain. The *red sphere* and *yellow sticks* indicate the Ca^2+^ ion and Tris bound on the posterior side, respectively. Water molecules are represented by crosses (no H or D atoms modeled) or lines. *D*, the active site is illustrated by *sticks* of the ligand (Rha and acetate at subsite −1 and +1) and the catalytic residues. The substrate (Rha-GlcA) in the X-ray structure (PDB ID: 7ESN) is superimposed (*magenta lines*). H and D atoms are colored *white* and *cyan*, respectively. The chemical structure of Rha-GlcA is presented in the *inset*.

**Figure 2:**
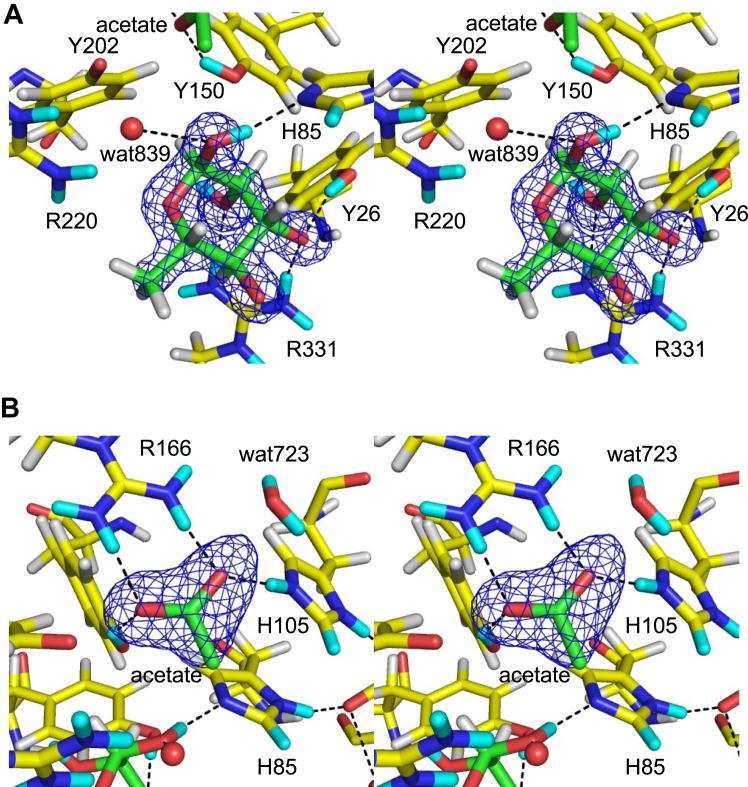
**Stereo view of Rha and acetate bound to the XN structure.***A*, Rha at subsite −1 with *mF*_o_-*DF*_c_ omit map (7.0σ). *B*, acetate ion at subsite +1 with *mF*_o_-*DF*_c_ omit map (4.0σ). X-ray electron density (XRED) maps are shown as *blue mesh*. H and D atoms are colored *white* and *cyan*, respectively. Hydrogen bonds are illustrated with *black dotted lines*.

The recombinant WT FoRham1 protein produced in *Pichia pastoris* was used for crystallization. H/D exchange was achieved by changing the solution of purified protein with deuterated buffer, and the crystals were grown using solutions prepared with heavy water. A crystal grown to approximately 1.5 mm^3^ in the presence of Rha was used for the multi-probe quantum beam diffraction experiments (Figs. 1*A* and S1). The resolutions of X-ray and neutron diffraction datasets collected at room temperature were 1.25 and 1.80 Å, respectively (Table 1). The resolution of the current X-ray data was improved over the previously reported data collected at a cryogenic temperature (1.40 Å, PDB ID: 7ESM). The non-hydrogen atoms were modeled according to the X-ray electron density (XRED) map. Joint XN structure refinement was successfully performed and completed with reasonable statistics for crystallography and protein stereochemistry (Table 1). In the final XN structure, 3130 H atoms, 894 D atoms, and 299 water molecules were modeled (Fig. 1*B*) based on the neutron scattering length density (NSLD) maps. The C-terminal polyhistidine and c-Myc tags were not modeled due to disorder as in the previous X-ray structure (20), whereas a high-mannose *N*-glycan at Asn247 was (Fig. 1*C*). We observed GlcNAc_2_-Man_5_ heptasaccharide in the XN structure (Fig. S2), whereas only tetrasaccharide (GlcNAc_2_-Man_2_) was previously observed in the high-resolution X-ray structure (1.05 Å; PDB ID: 7ESK) (20). Two monoatomic cations (Na^+^ and Ca^2+^) were observed at the same positions as those in the previous X-ray structures (20). A Tris molecule, derived from the reservoir solution for growing huge crystals, was bound on the surface of the posterior side (Fig. S3). The two cations and the Tris molecule were located away from the active site and did not participate in substrate-binding or catalysis. Rha and an acetate ion were bound at subsites −1 and +1, respectively, as observed in the previous X-ray structure of WT complexed with Rha (Figs. 1*D* and 2) (20).

**Table 1:** Crystallographic data collection and refinement statistics

Dataset	WT FoRham1 + Rha (XN structure at room temperature)	
Data collection	X-ray	Neutron
Beamline	KEK PF BL-5A	J-PARC MLF BL03
Wavelength (Å)	1.0000	2.28–6.19
Temperature	Room temperature	Room temperature
Space group	*P*2_1_2_1_2_1_	
Unit cell (Å)^a^	*a* = 57.78, *b* = 65.80, *c* = 108.95	
Resolution (Å)	43.42–1.25 (1.27–1.25)	19.82–1.80 (1.86–1.80)
Total reflections	733,688 (36,002)	306,096 (22,656)
Unique reflections	115,388 (5667)	38,826 (3825)
Completeness (%)	100.0 (100.0)	98.9 (98.0)
Redundancy	6.4 (6.4)	7.9 (5.9)
Mean *I*/σ(*I*)	15.0 (2.2)	8.4 (1.6)
*R*_*merge*_ (%)	5.9 (88.6)	25.7 (117.4)
*R*_*pim*_ (%)	2.5 (38.0)	9.6 (50.7)
CC_1/2_	(0.794)	(0.411)
Wilson B-factor (Å^2^)	14.07	
Refinement		
Resolution (Å)	43.42–1.25 (1.26–1.25)	19.8–1.80 (1.84–1.80)
No. of reflections	115,291	38,824
*R*_work_ (%)	14.7 (25.5)	16.2 (27.9)
*R*_free_ (%)	16.3 (24.0)	19.1 (30.4)
Number of atoms	7768	
Number of waters	299	
RMSD from ideal values		
Bond lengths (Å)	0.007	
Bond angles (°)	1.160	
Ramachandran plot (%)		
Favored	96.2	
Allowed	3.8	
Outlier	0	
PDB code	7YQS	

Values in parentheses are for the highest resolution shell.

a Values are determined using the X-ray diffraction data.

### Rha and acetate in the active site

**Figure 3:**
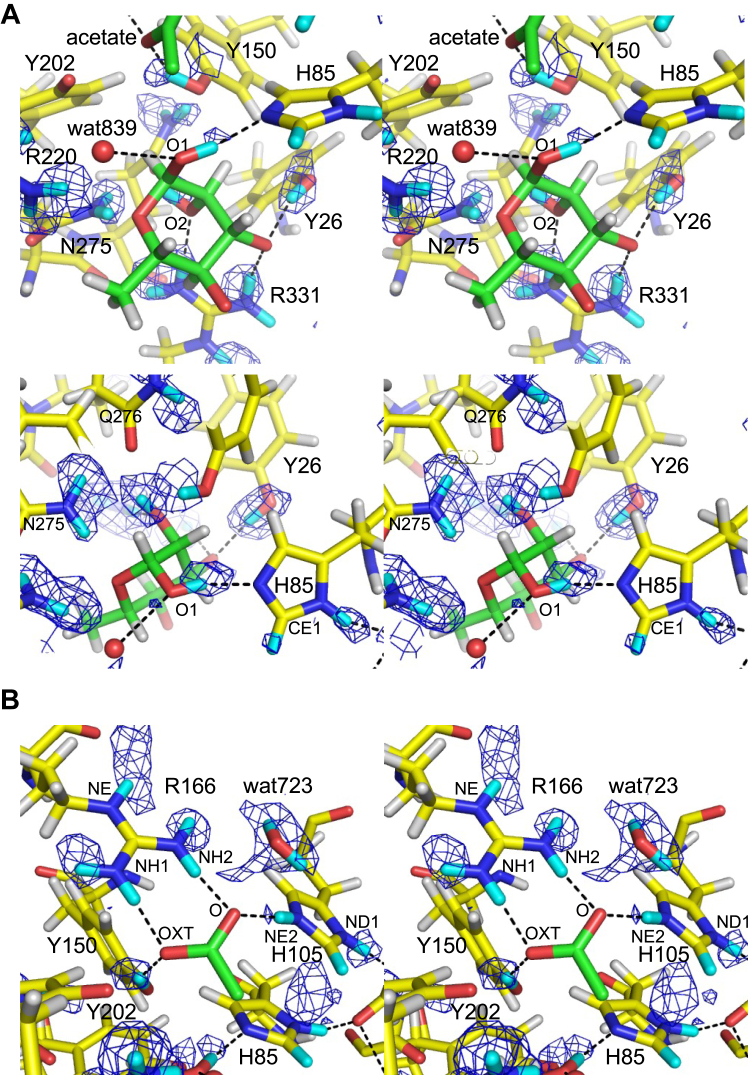
**Stereo view of the Rha and acetate binding sites.***A*, the Rha binding site centered at Rha (*upper panel*, 2.9σ) and His85 (*lower panel*, 2.2σ). *B*, acetate binding site (2.7σ). *mF*_o_-*DF*_c_ neutron scattering length density (NLSD) maps are shown as *blue mesh*. H and D atoms are colored *white* and *cyan*, respectively. Hydrogen bonds are illustrated with *black dotted lines*. The following atoms were excluded for map calculation: DO1 and DO2 of Rha; DH1 of Tyr26; DD1 and DE1 of His85; DD1, DE1 and DE2 of His105; DH of Tyr150; DE, DH11, DH12, DH21, and DH22 of Arg166; DE, DH11, DH12, DH21, and DH22 of Arg220; DD21 and DD22 of Asn275; DE21 and DE22 of Gln276; DE, DH11, DH12, DH21, and DH22 of Arg331; and D1 and D2 of wat723.

Figure 3*A* presents the NSLD map of Rha. Protonation near the hydroxy groups of Rha was determined by the density peaks that appeared *via* H/D exchange. A strong positive peak (D:H = 0.62:0.38) was observed near the OH atom of Tyr26. This D atom formed a hydrogen bond with the O3 atom of Rha. The O1 atom of Rha was located within the hydrogen bond distance to the NE2 atom of His85 and the OH atom of Tyr150. The D atom involved in the hydrogen bond between the O1 of Rha and NE2 of His85 was at a covalent bond distance to the O1 atom. The D:H occupancy ratio of this atom was 0.62:0.38. No positive peak was observed between the Rha O1 atom and the Tyr150 OH atom, whereas the OH atom of Tyr150 formed a hydrogen bond with acetate. The hydrogen bond between His85 and the O1 atom of Rha, which corresponds to the glycosidic bond oxygen, suggests that the catalytic proton donor for the bond cleavage is His85, not Tyr150. At the HE1/DE1 atom of His85, a positive peak (D:H = 0.60:0.40) was observed in the NSLD map (Fig. 3*A* lower panel and Fig. S4*A*), suggesting that H/D exchange occurred at the C–H bond (Supplementary Methods in Supporting Information and Fig. S5). The exchange of the HE1 atom of histidine residues with the DE1 atom occurred in the neutron crystallography of myoglobin (22, 23) and catalase (24). H/D exchange of the C–H bond was also observed in the guanine base of Z-DNA (25). Arg331 was hydrogen-bonded to the O2 and O3 atoms in Rha (Fig. 3*A* upper panel). Positive peaks near the NH1 and NH2 atoms of Arg331 and the O2 atom of Rha were observed, and the D:H ratios of these atoms were 0.65:0.35, 0.67:0.33, and 0.65:0.35, respectively.

**Figure 4:**
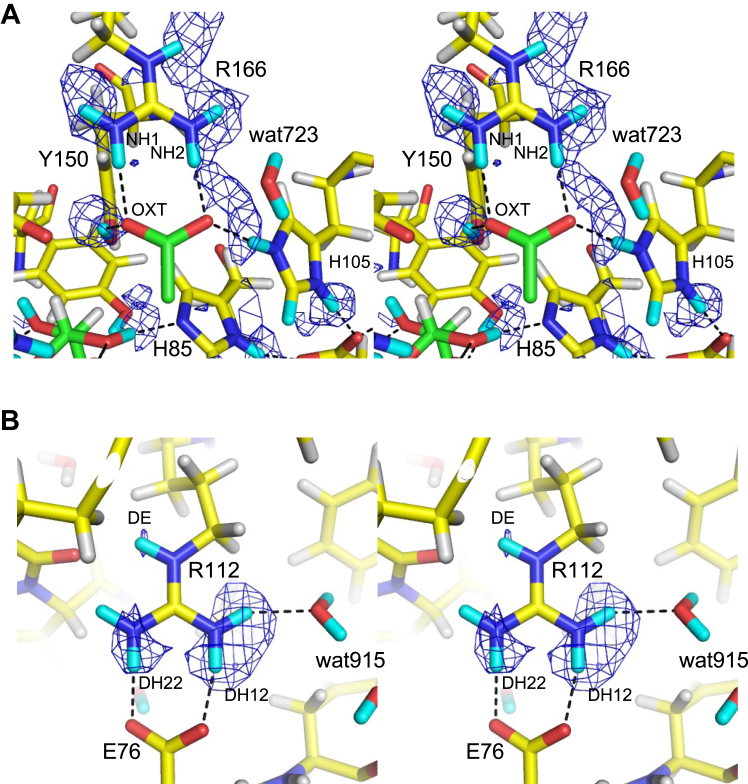
**Comparison of two Arg-carboxylate salt bridge interactions illustrated by stereo views.***A*, acetate-binding site. This map is at a contour level of 2.2σ, lower than that in Figure 3*B* (2.9σ). The following atoms were excluded from the map calculation: DO1 and DO2 of Rha, DD1 and DE1 of His85, DD1, DE1, and DE2 of His105, DH of Tyr150, DE, DH11, DH12, DH21, and DH22 of Arg166. *B*, Arg112 and Glu76 with a map contoured at 2.9σ. DE, DH11, DH12, DH21, and DH22 of Arg112 were excluded from the map calculation. *mF*_o_-*DF*_c_ NLSD maps are shown as *blue mesh*. H and D atoms are colored *white* and *cyan*, respectively. Hydrogen bonds are represented with *black dotted lines*.

The position of acetate corresponds to GlcA carboxylate at subsite +1 (Fig. 1*D*). Acetate was bound within the hydrogen bond distances to the NE2 atom of His105, OH atom of Tyr150, NH1 and NH2 atoms of Arg166, and OH atom of Tyr202 (Fig. 3*B*). A strong positive peak (D:H = 0.65:0.35) was observed near the OH atom of Tyr150. This D atom formed hydrogen bonds with the OXT atom of acetate, indicating that Tyr150 was deuterated. No positive peak was observed between acetate and Tyr202, indicating no hydrogen bond between the OH atom of Tyr202 and the OXT atom of acetate (distance = 2.6 Å). No strong positive peaks were observed between the acetate and side chain nitrogen atoms of Arg166 (NH1 and NH2) at 2.2 to 2.9σ levels (Figs. 4*A* and S4*C*). In contrast, positive peaks of DH11, DH21, and DE atoms of Arg166 were observed (Fig. S4*C*). Yonezawa *et al.* (26) proposed that Arg52 in the photoactive yellow protein, which is hydrogen-bonded with tyrosine and threonine side chains, adopts an electrically neutral form. However, we inferred that the Arg166 side chain was positively charged due to the apparent presence of a salt bridge with acetate. When the H/D exchange was incomplete, the negative and positive peak contributions of the H and D atoms were canceled to yield no or weak peaks in the NSLD maps (22). The calculated D:H occupancy ratios of the atoms between the acetate and the NH1 and NH2 atoms were 0.55:0.45 and 0.49:0.51, respectively. The salt-bridged local structure of Arg166-acetate is similar to that of the Arg–Glu/Asp interactions in proteins; therefore, we examined the NSLD map around the salt bridge interaction between Arg112 and Glu76 for comparison (Fig. 4*B*). In the Arg112 side chain, positive peaks were observed near the NH1 and NH2 atoms at 2.9σ contour level (D:H = 0.73:0.27 and 0.67:0.33 for DH12 and DH22 atoms, respectively), indicating that H atoms were efficiently exchanged with D atoms in this area. The D:H occupancy ratios in refined structures of joint XN crystallography aid in the interpretation of the hydrogen bond strength and local stability (27). Therefore, the lower D:H occupancy ratio of the atoms between Arg166 and acetate compared to the reference Arg–Glu interaction may indicate a strong salt bridge interaction, resulting in an incomplete H/D exchange.

Regarding the side chain of His105, H/D exchange also occurred at the C–H bond of CE1 (DE1: HE1 = 0.27:0.73), similar to His85 (Supplementary Methods in Supporting Information and Fig. S5). Furthermore, between the O atom of acetate and the NE2 atom of His105, a weak positive peak near His105 (D:H = 0.52:0.48) was observed (Figs. 3*B* and S4). Moreover, a positive peak was also observed near the ND1 atom of His105; therefore, this histidine had a positive charge, as it was doubly deuterated. The positive peak near the NE2 atom of His105, weaker than the ND1 atom, might also be due to the strong salt bridge interaction with acetate. The positively charged side chains of Arg166 and His105 in subsite +1 facilitate substrate-binding and stabilize the negative charge of GlcA carboxylate.

### Mutational analysis

**Figure 5:**
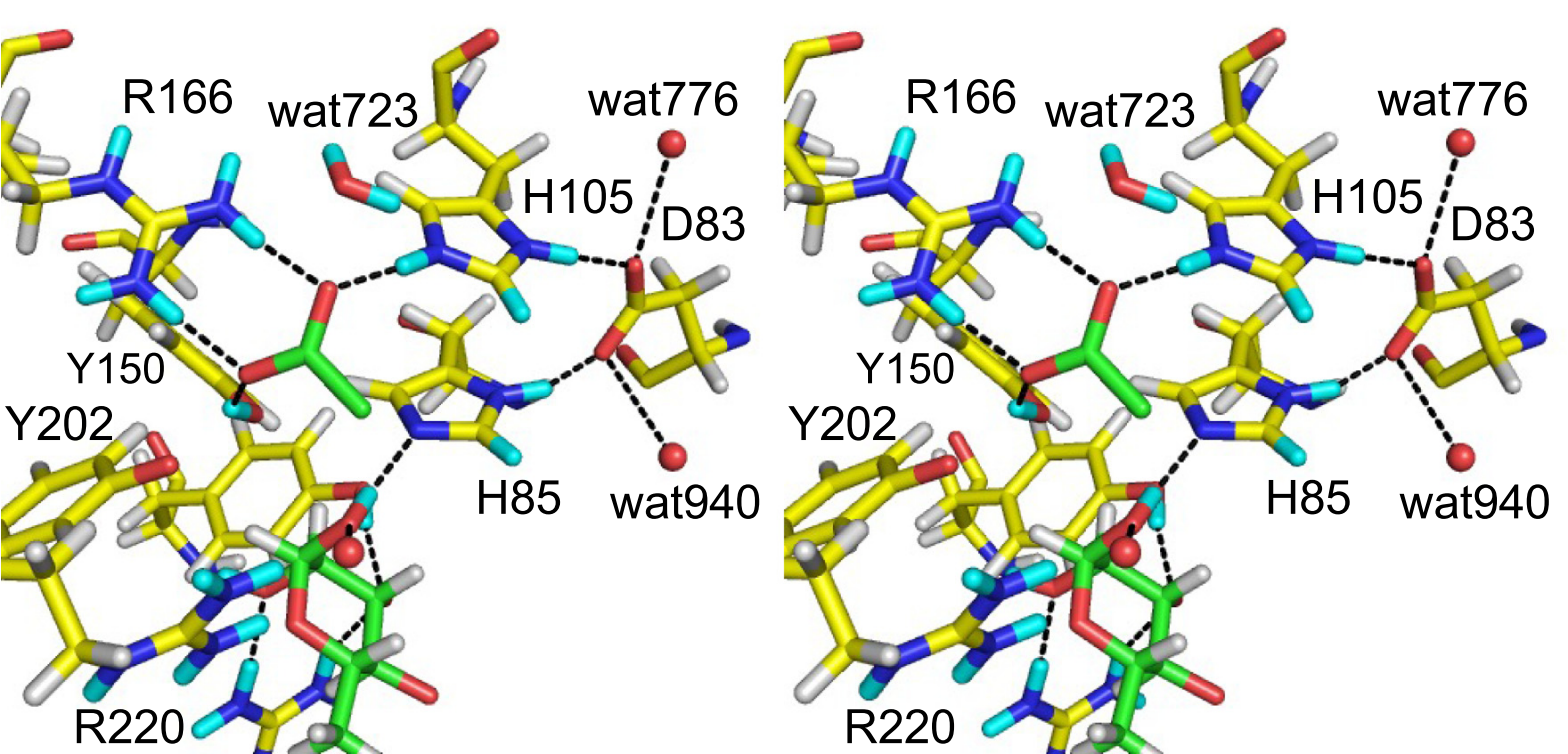
**Stereo view of the catalytic site, including Asp83.** D and H atoms (*cyan* and *white*) determined *via* joint XN crystallography are presented.

**Figure 6:**
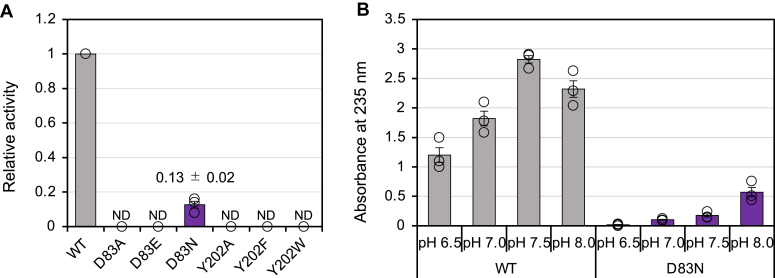
**Enzymatic activities of WT FoRham1 and mutants for 1% GA.***A*, relative activities of FoRham1 and the mutant enzymes in 50 mM HEPES–NaOH (pH 7.0) at 30 °C. *B*, the activities of WT FoRham1 and D83N in 50 mM MOPS–NaOH (pH 6.5–8.0) at 30 °C. The produced ΔGlcA generated at the non-reducing end of GA side chains was quantified at 235 nm absorbance.

In our previous report, site-directed mutants of several residues, including His85, Arg166, His105, and Tyr150, in the active site of FoRham1 were analyzed, and these residues were important for enzyme activity (20). In this study, we focused on Tyr202 near the acetate ion (Fig. 3*B*). Asp83 may also play an important role because it forms hydrogen bonds with the catalytically essential “double histidine” residues, His85 and His105 (Fig. 5). Therefore, we prepared site-directed mutants of Asp83 and Tyr202. The activities of D83A, D83E, Y202A, Y202F, and Y202W toward GA were undetected, and D83N retained 13% of its activity compared with the WT (Fig. 6*A*). In contrast to the WT, with an optimal pH of 7.5, the activity of D83N increased at a higher pH (Fig. 6*B*). This result suggests that Asp83 in the deprotonated state holds the two catalytic histidine residues at precise positions and controls their charge states, thereby supporting enzyme catalysis. Moreover, the side-chain hydroxy group of Tyr202 was essential for this activity. As there was no positive peak near the OH atom of Tyr202 (Fig. 3*A*), we presumed that this residue was deprotonated. The positively charged environment between Arg166 and Arg220 may stabilize the deprotonated (negatively charged) state of Tyr202 and lower its p*K*_a_ (Fig. 5). The detailed function of Tyr202 in enzyme catalysis remains elusive; however, its deprotonated side chain may maintain a charge balance in the active site with the two nearby arginine residues (Arg166 and Arg220).

### His–His–Asp triad and possible H/D exchange mechanism

**Figure 7:**
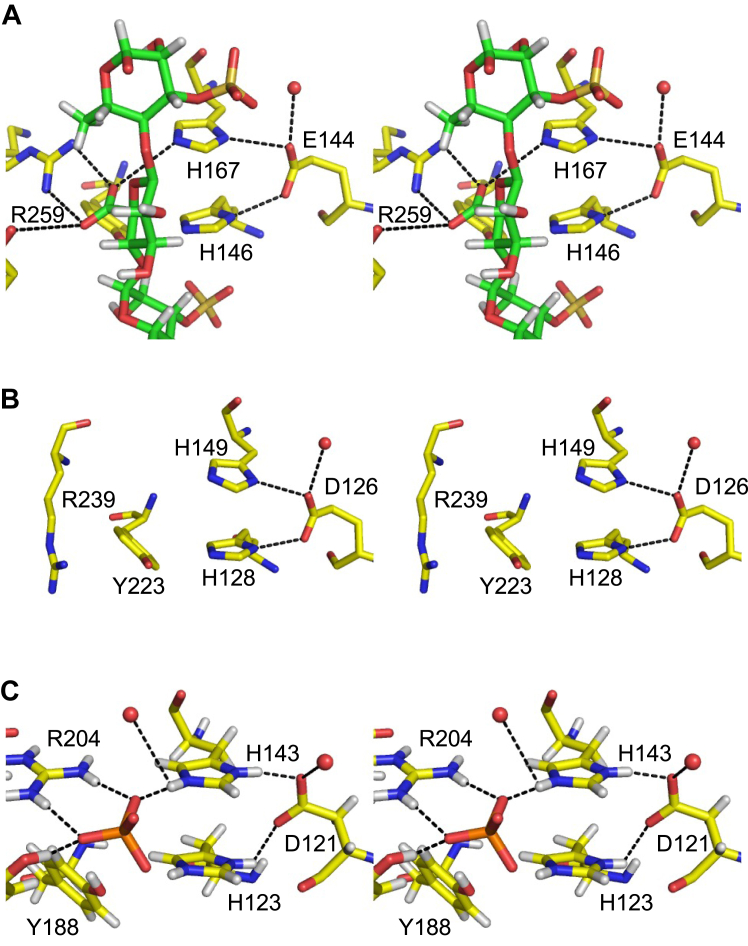
**Active sites of ulvan lyases belonging to PL24 and PL25.***A*, PL24 LOR_107 from *Alteromonas* sp. strain LOR (PDB ID: 6BYT) complexed with an ulvan tetrasaccharide substrate (*green*). *B*, PL24 Uly1 from *Catenovulum maritimum* (PDB ID: 7DRQ). *C*, PL25 PLSV_3936 from *Pseudoalteromonas* sp. strain PLSV (PDB ID: 5UAM), complexed with sulfate (*orange*). In *A* and *C*, hydrogen atoms modeled in the deposited coordinate file based on the X-ray crystal structure are presented.

The double histidine motif is conserved in the PL42 family and other PL families (20). Here, we found that Asp83, which bridges the histidine residues behind the active site, is also essential for enzyme activity. The His–His–Asp triad motif is well-conserved in bacterial ulvan lyases belonging to PL24 and PL25 families (Fig. 7) (28, 29, 30). The carboxy group of Asp83 was hydrogen-bonded to the ND1 atoms of His85 and His105 (Fig. 5). The positive peaks were observed near the ND1 atoms of His85 and His105, and the D:H occupancy ratio of DD1/HD1 atoms in His85 and His105 were 0.54:0.46 and 0.68:0.32, respectively, indicating that the hydrogen atoms in both histidine residues were deuterated (Fig. S4, *A* and *B*). Two water molecules were hydrogen-bonded to Asp83, and a water molecule (wat723) was around the NE2 atom of His105 (Fig. 5). A possible H/D exchange mechanism of His105 and His85 by solvent molecules is summarized in Fig. S6. The FoRham1 crystal was obtained in a mixture of equal volumes of pD 8.0 protein solution and pD 8.5 reservoir solution (see Experimental procedures). Since D_2_O concentration is significantly higher than that of D_3_O^+^ and OD^−^, the mechanism with which D_2_O acts as an H^+^ acceptor and D^+^ donor is presented. The frequent interconversion of His85 protonation with nearby hydrogens (or deuterium) is suitable for its catalytic role as a general base/acid.

### Comparison with cryogenic structure

**Figure 8:**
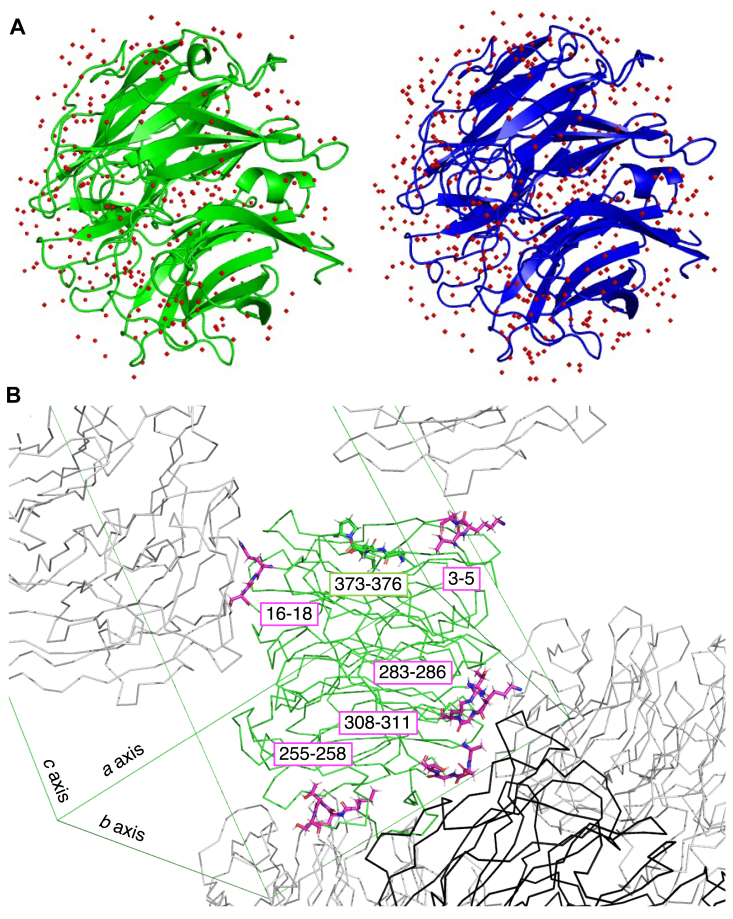
**Effect of data collection temperature.***A*, water molecules (*red spheres*) observed in the structures at room temperature (*green*, *left*) and cryogenic temperature (*blue*, *right*) are shown. *B*, crystal packing of the room temperature structure (*green*). Symmetry-related molecules are colored *grey* or *black*. The displaced regions in the cryogenic temperature structures are shown as *sticks*. Regions involved in the crystal packing and those not involved are colored *magenta* and *green*, respectively.

To elucidate temperature-dependent effects on the enzyme structure, we determined the cryogenic X-ray crystal structure of FoRham1 complexed with Rha. A protein sample from the same purification lot used for the room temperature crystallography was crystallized under similar conditions after H/D exchange and cryo-cooled in liquid nitrogen without using an additional cryoprotectant (Fig. S7). The X-ray structure was determined at 1.06 Å resolution (Table S1). The resolution was improved over the previously determined cryogenic structure (1.40 Å, PDB ID: 7ESM) (20). As expected, the Wilson B-factor of the cryogenic temperature crystal was significantly lower than that of the room temperature crystal (Table 1). In the final structure, 458 water molecules were included, 159 more than the waters modeled in the room temperature structure. The number of water observed on the protein surface was significantly increased by cryocooling (Fig. 8*A*). In the cryogenic structure, 12 amino acids were modeled with alternative conformations of the side chain, while the room temperature structure has no residues with alternative conformations (Table 2 and Fig. S8). This was an unexpected structural feature of the FoRham1 crystal, as protein crystal structures generally adopt less variable conformations by cryocooling (31). The 12 residues that exhibited alternative side chain conformations are not involved in catalysis or substrate-binding, suggesting that the changes in side chain conformations by cryocooling are not relevant to enzyme function. Comparing the cell constants of the room and cryogenic temperature crystals, the latter has a 4.04% smaller unit cell volume than the former, mainly due to the shrinking of the *a* axis by 1.47 Å (Table 2). The protein volume of the cryogenic temperature structure is about 1.0% smaller than that of the room temperature structure (Table 2). These changes in crystallographic size by cryocooling are consistent with the general property of protein crystals in that the decrease in unit cell volume exceeds the decrease in protein volume (31).

**Table 2:** Comparison of the crystal lattice, protein volume, and side chain conformations between the room and cryogenic temperature structures

Features	XN structure at room temperature	X-ray structure at cryogenic temperature	Cryogenic changes (%)
Cell constants (Å)	*a* = 57.78, *b* = 65.80, *c* = 108.95	*a* = 56.31, *b* = 65.23, *c* = 108.22	−2.54, −0.87, −0.67 (*a*, *b*, *c*)
Unit cell volume (Å^3^)	414,220	397,503	−4.04
Protein volume (Å^3^)^a^	68,367	67,659	−1.04
Alternative conformation residues	None	E44, K115, D129, S170, S172, S183, M192, I194, L294, N315, N318, K386	–

a Calculated using 3V website (http://3vee.molmovdb.org) (49).

Superimposition of the room and cryogenic temperature structures shows significant deviations (>0.4 Å for Cα atom) in six regions (Fig. S9). Among them, five regions are involved in the crystal packing (Fig. 8*B*), and none of these regions include the residues with side chain alternative conformations. No significant difference between the room and cryogenic temperature structures was observed in the active site, indicating that the data collection temperature did not affect our interpretation of the crystallographic structure.

## Discussion

**Figure 9:**
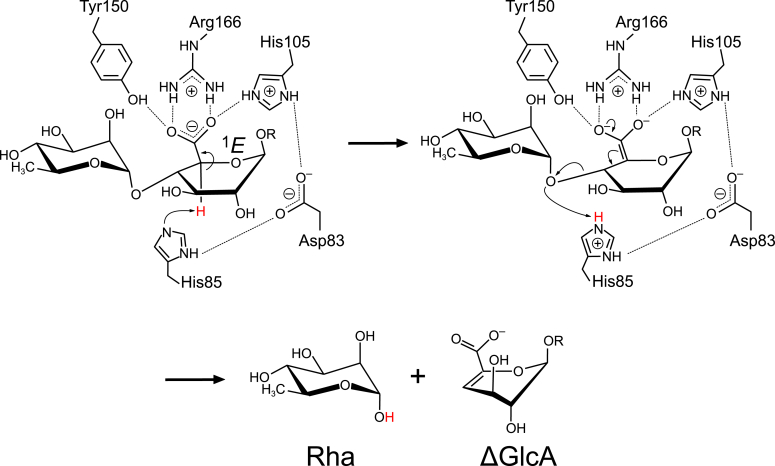
**Proposed reaction mechanism of FoRham1.** The two-step *syn*-elimination mechanism is presented with catalytically important residues. R = H or another sugar residue.

The updated catalytic mechanism for FoRham1 proposed in this study is illustrated in Figure 9. This mechanism is based on a stepwise *syn*-elimination pathway, often proposed for metal-independent PLs (32, 33). The function of Arg166 as a charge neutralizer is enhanced by the side chains of the positively charged His105 and protonated Tyr150. The unique environment of the GlcA carboxylate with four hydrogen bond donors (two from Arg166 and one each from His105 and Tyr150) increases the reactivity of the C5 atom and stabilizes the oxyanion of an “electron sink” that is formed after proton abstraction. His85 functions as a catalytic base in the first step because the NE2 atom is deprotonated, as revealed in this study. A distorted envelope conformation (^1^*E*) of the GlcA pyranose ring alleviates the steric hindrance around the H5 atom and brings the hydrogen atom closer to His85 (Fig. 1*D*) (20). In the second step, His85 functions as an acid to donate a proton to the glycosidic bond oxygen. In this study, we observed a hydrogen bond between the deprotonated His85 and deuterated O1 atom of Rha (Fig. 3*A*). Rha is a reaction product; therefore, the XN crystal structure mimics the state after the glycosidic bond cleavage at subsite −1 (Fig. 9). In the catalytic cycle, the deprotonated His85 will be able to act as the catalytic base when the next substrate enters the active site. His85 is connected to His105 and water molecules through hydrogen bonds *via* Asp83. This hydrogen-bond network possibly modulates the p*K*_a_ of His85 at each catalytic step and supports the dual function of base/acid catalysis.

Joint XN crystallography of FoRham1 with high-resolution data was used to visualize the positions of the D and H atoms in the crystal complex with the reaction product, Rha. Fortunately, an acetate ion was bound to the same position as the carboxylate group of GlcA in subsite +1 (Fig. 1*D*). Thus, the acetate mimicked the carboxylate group of GlcA and illustrated the unique hydrogen-rich environment at this site. The direct observation of the D atoms around the O1 atom of Rha excluded Tyr150 from the catalytic residue candidates in the glycosidic bond cleavage, providing evidence that His85 is the catalytic acid. The deuterated state of Tyr150 suggests that it is not likely the catalytic base for the first reaction step. This contrasts with the PL25 ulvan lyase PLSV_3936, where Tyr188 (equivalent to Tyr150) was suggested to function as the catalytic base (30). The “second” histidine, His105, supports the charge neutralizer, Arg166, and modulates the catalytic base/acid functions of His85 through a hydrogen bond network. Asp83 seems to play a pivotal role in controlling the protonation of the double histidine residues, and the His–His–Asp triad motif is conserved among the PL42, PL24, and PL25 families. The hydrogen bond chain between the histidine residues of FoRham1 is reminiscent of the “Newton’s cradle” proton relay between the catalytic base and acid residues of an anomer-inverting glycoside hydrolase, PcCel45A (34). Further studies on visualizing hydrogen/deuterium atoms in numerous carbohydrate-active enzymes will reveal their precise regulatory mechanisms in intricate three-dimensional structures.

## Experimental procedures

### Preparation of huge protein crystals

The recombinant protein produced in *P*. *pastoris* with a C-terminal 6× histidine and c-Myc tag was purified as previously described (20). In a previous cryogenic X-ray diffraction experiment, crystals of WT FoRham1 complexed with Rha were obtained *via* the sitting-drop vapor diffusion method with a 96-well crystallization plate. The crystallization drop was made by mixing the protein solution (24 mg/ml protein, 20 mM Tris-HCl pH 8.0, and 0.1 M Rha) with the reservoir solution of PEG Rx HT #66 (0.1 M Bicine pH 8.5, 10% [v/v] 2-propanol, and 30% [w/v] PEG1500) purchased from Hampton research. The crystals did not grow in homemade crystallization solutions (20); therefore, the crystallization conditions were thoroughly re-examined to obtain huge crystals for neutron diffraction experiments. Reagents with heavy water (99.9% D_2_O) were used for crystallization to facilitate the H/D exchange of the protein and reduce incoherent background scattering from H atoms in the diffraction pattern. The purified protein solution was exchanged with 20 mM Tris pD 8.0 buffer using Amicon Ultracel-10K and concentrated to 24 mg/ml. A large-scale sitting-drop vapor diffusion method was adopted using a Falcon 60 × 15 mm center well organ culture dish (Corning Inc) (24). In the outside well of the culture dish, 2 ml of reservoir solution consisting of 0.1 M Tris pD 8.5, 0.1 M Rha, and 33.0% (w/v) PEG1500 in heavy water was poured. A stock solution of 1 M Rha, dissolved in heavy water, was used to prepare the reservoir solution. A siliconized glass cover slide was placed in the center well of the organ culture dish. A drop prepared by mixing 100 μl of protein solution and 100 μl of reservoir solution was placed on a cover slide. The culture dish was closed with a lid using high-vacuum grease (Dow Toray Co, Ltd) as a sealer and incubated at 20 °C for approximately 10 weeks until the crystals grew to >1 mm^3^.

### Neutron and X-ray diffraction experiments at room temperature for the joint XN crystallography

The crystal of WT FoRham1 complexed with Rha and 20 μl of the reservoir solution were sealed in a quartz capillary with 3.5 mm Φ and 0.01 mm thickness (Hilgenberg GmbH) with a custom-made stainless-steel magnet base (Fig. S1). Time-of-flight neutron diffraction experiments were performed using BL03 IBARAKI biological crystal diffractometer (iBIX) in the Materials and Life Science Experimental Facility (MLF) of Japan Proton Accelerator Research Complex (J-PARC) (35, 36, 37) with 34 two-dimensional position-sensitive detectors using a scintillator sheet and wavelength-shifting fiber at room temperature (38). The accelerator power of the proton beam for the spallation neutron source was 600 kW. A neutron diffraction dataset was collected using a circular beam of 3 mm diameter and a selected neutron wavelength of 2.28 to 6.19 Å. The capillary sample was placed on a three-axes goniometer and exposed to 736,000 pulsed neutrons for each crystal orientation. In total, 34 goniometer settings were selected to collect the entire dataset required for structure refinement. Incoherent neutron scattering data from a 4.8-mm vanadium sphere were collected using the same neutron wavelength range as the protein crystal. This procedure was performed to correct the variance in the detection efficiency of pixels within one detector, the difference in neutron beam intensities by wavelength, and the difference in detection efficiency by wavelength. Data reduction was performed using STARGazer 3.4.3 (39), which employs a profile-fitting method for peak integration (40). Data statistics were calculated using the unit cell constants determined *via* X-ray diffraction.

After neutron diffraction data measurements, the same crystal was used for the synchrotron X-ray diffraction experiment on BL-5A at the Photon Factory of the High Energy Accelerator Research Institute (KEK). The crystal was exposed to an X-ray beam of 1.0 Å of 200 μm × 200 μm at 10% of the maximum intensity at room temperature. Diffraction images of 180 frames were obtained using the oscillation method with 1.0° steps and 1.0-s exposure per frame. The diffraction images were processed using XDS (41) and statistics for the data collection were calculated using AIMLESS (42).

### Joint XN refinement

Joint XN refinement was performed using PHENIX 1.17.1_3660 (43, 44) and Coot (45). The X-ray and neutron diffraction datasets were merged into an MTZ format file using the reflection tool in PHENIX. Five percent of the reflections commonly existing in both datasets were randomly assigned as a test dataset for cross-validation. The initial structure model was solved *via* the molecular replacement method using X-ray data and the atomic coordinates of WT FoRham1 (PDB ID: 7ESK), from which the solvent water molecules and ligands were removed. After several cycles of the atomic coordinates and temperature factor refinement using the X-ray intensity data, oxygen atoms of water molecules, Na^+^, Ca^2+^, and C and O atoms of Tris, Rha, and acetate were placed in the model, and the X-ray refinement was repeated. Acetate was not used during the protein expression, purification, and crystallization. The acetate ion was probably derived from secreted metabolites of the *P. pastoris* culture. After several cycles of joint XN refinement, NSLD peaks that seem derived from H and D atoms were observed in the *mF*_o_-*DF*_c_ map. H and D atoms were placed in the model using phenix.readyset. The exchangeable hydrogen site atoms were treated as disordered (multiple) models of H and D atoms, and the initial occupancies were set at 0.5/0.5. Then, exchangeable hydrogen site atoms other than those in the main chains were removed from the atomic coordinates. The coordinates and temperature factors of all atoms were refined using this model. The occupancies of only exchangeable hydrogen atom sites were also refined. Since *R*_work_ and *R*_free_ between the intensity data of neutron and the model decreased by approximately 9% before and after adding H and D atoms, H and D atom information was included in the neutron intensity data. The H and D atoms at exchangeable sites of the protein side chains, solvent water molecules, and ligands were manually added to the atomic coordinates and refined. H and D atoms at exchangeable hydrogen sites were added when a peak was observed in the *mF*_o_-*DF*_c_ NSLD map, or non-hydrogen atoms bonded to exchangeable hydrogens were observed in the XRED map. The protein solution was exchanged with a heavy water solution after protein purification, and the heavy water solution was also used for crystallization; therefore, water was regarded as D_2_O, and D atoms with an occupancy of 1.0 were manually added. The procedures for modeling and the refinements were repeated until all observed H and D atoms were included in the model. We confirmed whether the residual densities of the *mF*_o_-*DF*_c_ NSLD map were reduced by adding H and D atoms. When negative peaks were confirmed in D atoms of water molecules, the initial occupancies were set at 0.5, and the atomic coordinates, temperature factors, and occupancies were refined. The procedure for determining the CE1 atom protonation of His85 and His105 is described in Supplementary Methods and Table S2 in Supporting Information. All molecular figures were drawn using PyMOL (Schrödinger LLC).

### Cryogenic crystallography of WT complexed with Rha

The purified protein solution was exchanged with 20 mM Tris pD 8.0 buffer and concentrated to 24 mg/ml in the same way as huge crystals. A sitting-drop vapor diffusion method was adopted using a 24-well Cryschem Plate (Hampton Research). In the outside well of the plate, 400 μl of reservoir solution consisting of 0.1 M Tris pD 8.5, 0.1 M Rha, and 36.0% (w/v) PEG1500 in heavy water was poured. A stock solution of 1 M Rha, dissolved in heavy water, was used to prepare the reservoir solution. A drop prepared by mixing 4 μl of the protein solution and 4 μl of the reservoir solution was placed in a well. The plate was closed with transparent tape as a sealer and incubated at 20 °C. Because PEG1500 acts as a cryoprotectant, no cryoprotectant treatment was performed. Crystals were scooped by a cryoloop (Hampton Research) and cryo-cooled by dipping into liquid nitrogen. The synchrotron X-ray diffraction experiment was performed on AR-NE3A at the Photon Factory. The crystal was exposed to an X-ray beam of 1.0 Å wavelength with a 100 μm diameter at 100% of the maximum intensity at cryogenic temperature (100 K). Diffraction images of 360 frames were obtained using the oscillation method with 1.0° steps and 1.0-s exposure per frame. The data processing and crystallographic refinement were performed using the same program version that was used for the joint XN crystallography at room temperature. H and D atoms were removed from the room temperature structure for comparison with the cryogenic structure.

### Site-directed mutant analysis

Site-directed mutagenesis to construct the variants was performed using the PrimeSTAR mutagenesis basal kit (Takara Bio Inc) with the pPICZαA vector containing the mature *Forham1* gene as the template. The primers used here are presented in Table S3. Recombinant enzymes expressed in *P. pastoris* were purified and assayed as previously described (20). GA (Lot No. 120M0035V) was purchased from Sigma-Aldrich Co. Enzymatic activities of WT and mutants were measured using GA (1%) as the substrate (n = 3). The enzymes (0.2 μM) were incubated with the substrate in 50 mM HEPES–NaOH (pH 7.0) or 50 mM MOPS–NaOH (pH 6.5–8.0) at 30 °C for 10 min. The reaction mixture was then boiled for 3 min to inactivate the enzyme. Finally, the amount of ΔGlcA generated at the non-reducing end of GA side chains was measured by absorbance at 235 nm.

## Data availability

The data underlying this article are available upon request to the corresponding author. The atomic coordinates and intensity datasets of X-ray and neutron diffraction are available at the Protein Data Bank (PDB codes 7YQS and 8I4D for room temperature XN and cryogenic X-ray structures, respectively).

## Supporting information

This article contains supporting information (46, 47, 48).

## Conflict of interest

The authors declare that they have no conflict of interest with the contents of this article.
