# Supplementary material for: Charge neutralization and β-elimination cleavage mechanism of family 42 L-rhamnose-α-1,4-D-glucuronate lyase revealed using neutron crystallography
Source: J Biol Chem. 2024 Feb 19;300(3):105774. doi: 10.1016/j.jbc.2024.105774 (PMC10951650; doi:10.1016/j.jbc.2024.105774)
Supplement: Supporting information [file mmc1.docx]

**Supporting Information**

**Charge neutralization and β-elimination cleavage mechanism of family 42 L-rhamnose-α-1,4-D-glucuronate lyase revealed using neutron crystallography**

Naomine Yano, Tatsuya Kondo, Katsuhiro Kusaka, Takatoshi Arakawa, Tatsuji Sakamoto, and Shinya Fushinobu

- Supplementary Methods
- Supplementary Figures S1–S9
- Supplementary Tables S1–S3
- Supplementary References

**Supplementary Methods**

*Determination of His85 and His105 protonation/deuteration at CE1 atom*

Atomic coordinate (PDB format) files of the following states were prepared to determine the protonation of the CE1 atoms of His85 and His105: (a) a file without HE1 atom, (b) a file with HE1 atom of 1.0 occupancy, and (c) a file with disordered (multiple) models of HE1 and DE1 atoms with initial occupancies of 0.5:0.5. While atomic coordinates and temperature factors were refined for states (a) and (b), occupancies were additionally refined for state (c). Then, peak heights of *mF*_o_-*DF*_c_ NSLD maps at the HE1 atom positions of the three states were compared. The temperature factors of the His side chains of states (b) and (c) were also compared (Table S2).

In the *mF*_o_-*DF*_c_ NSLD map of state (a), a weak but significant positive peak was observed at the position corresponding to HE1 (or DE1) of His85 (Fig. S4*A*). In state (b), a positive peak was observed at the HE1 atom of His85 in the *mF*_o_-*DF*_c_ NSLD map (Fig. S5, occupancy of HE1 = 1.0). The neutron scattering length of the H atom is negative (-3.7 fm) (1). Therefore, if the actual occupancy of HE1 is < 1.0, a positive peak should be observed in the *mF*_o_-*DF*_c_ NSLD map, and the temperature factor of HE1 be higher than those of the surrounding atoms to compensate for the occupancy discrepancy. The refined temperature factor of HE1 of His85 of state (b) was 46.22 Å^2^, which was larger than the other atoms in the same His side chain by approximately 17 Å^2^ (Table S2). In state (c), the refined occupancy ratio of HE1:DE1 was 0.40:0.60, and the refined temperature factors (~29 Å^2^ for HE1 and DE1) were comparable to those of surrounding atoms. In the *mF*_o_-*DF*_c_ NSLD map of state (c), no peak was observed at the HE1/DE1 atom position. Therefore, we concluded that approximately 60% of the HE1 atoms bound to CE1 in His85 were exchanged with DE1.

At the position corresponding to HE1 (or DE1) of His105, positive and negative peaks were not observed in the *mF*_o_-*DF*_c_ NSLD omit map of state (a). In state (b), a weak positive peak was observed at the HE1 atom of His105 in the *mF*_o_-*DF*_c_ NSLD map (Fig. S5, occupancy of HE1 = 1.0). The refined temperature factor of HE1 of His105 of state (b) was 37.73 Å^2^, which was larger than the other atoms in the same His side chain by over 10 Å^2^ (Table S2). In state (c), the refined occupancy ratio of HE1:DE1 of His105 was 0.73:0.27, and the refined temperature factors (~30 Å^2^ for HE1 and DE1) were comparable to those of surrounding atoms. In the *mF*_o_-*DF*_c_ NSLD map of state (c), no peak was observed at the atom position of HE1/DE1. Therefore, we concluded that approximately 27% of the HE1 atoms bound to CE1 in His105 were exchanged with DE1.

**Supplementary Figures**

**Figure S1.** **Photographs of the huge crystal.** *A*, The crystallization drop. The initial drop volume was 200 μL. The crystal used for the diffraction experiments (Fig. 1*A*) is circled. *B*, The crystal was sealed in a quartz capillary.

**Figure S2. *N*-glycan observed in the XN structure.** *A*, Stereo view of the heptasaccharide (GlcNAc_2_-Man_5_) linked to Asn247 with 2*mF*_o_-*DF*_c_ X-ray electron density map (1.0σ, blue mesh). *B*, The stereo view of Man_3_ at the α-1,6 branch with *mF*_o_-*DF*_c_ omit X-ray electron density map (2.7σ, blue mesh). *C*, Schematic drawing of a typical high-mannose (oligomannose) *N*-glycan structure (2). Tetrasaccharide (GlcNAc_2_-Man_2_) and heptasaccharide observed in an X-ray and the joint XN structures are indicated, respectively. The glycan structure is represented using the Symbol Nomenclature for Glycans (SNFG) (3).

**Figure S3.** **Stereo view of a Tris molecule bound to the XN structure.** 2*mF*_o_-*DF*_c_ XRED map (blue mesh, 1.5σ) is shown. The H and D atoms are colored white and cyan, respectively. Hydrogen bonds are illustrated with black dotted lines. The Tris molecule is located at the interface of symmetry-related FoRham1 molecules. Residues from the symmetry-related molecule are indicated with prime symbols (G196’ and D197’).

**Figure S4.** **Stereo view of the key residue sidechains.** *A*, His85, *B*, His105, and *C*, Arg166 are shown with *mF*_o_-*DF*_c_ NSLD map at multiple counter levels. Maps at counter levels of 2.9σ (blue), 2.5σ (red), and 2.2σ (black) are illustrated. The following atoms were excluded from map calculation: DO1 and DO2 of Rha, DD1 and DE1 of His85, DD1, DE1, and DE2 of His105, DH of Tyr150, DE, DH11, DH12, DH21, and DH22 of Arg166.

**Figure S5.** **Stereo view of the side chain of His85 and His105 for determining CE1 atom protonation.** A *mF*_o_-*DF*_c_ NSLD map at a 2.9σ (blue mesh) counter level was calculated from the state (b) model, including the HE1 atom (occupancy = 1.0). The details are described in Supplementary Methods.

**Figure S6.** **Possible H/D exchange mechanism.** The mechanism of HD1 to DD1 in His105 (*A*), HE2 to DE2 in His105 (*B*), and HD1 to DD1 in His85 (*C*) are shown. *A*, HD1 of His105 initially moves to a solvent D2O molecule via OD2 of Asp83, producing HD_2_O^+^. D_2_O replaces the solvent position, and a D atom moves back to ND1 of His105 via Asp83. *B*, HE2 of His105 moves to a solvent D_2_O molecule via acetate oxygen, and a D atom moves back from a solvent D_2_O molecule to NE2 of His105 via acetate. *C*, Since a high-concentration stock solution of Rha dissolved in D_2_O was used for crystallization (see Experimental Procedures), the HO1 hydrogen atom on the anomeric C1 carbon of Rha is assumed to be fully deuterated via mutarotation. Finally, HD1 of His85 moves to a solvent D_2_O molecule via Asp83, and a D atom moves back from the solvent D_2_O molecule to ND1 of His85 via Asp83.

**Figure S7.** **Photographs of the crystals used for the experiment at cryogenic temperature.** *A*, The crystallization drop. The initial drop volume was 8 μL. *B*, The crystal during X-ray diffraction data collection.

**Figure S8.** **Comparison of electron density maps of the amino acid side chains.** Seven residues out of 12 residues exhibiting alternative conformations in the cryogenic temperature structure are selected. The room and cryogenic temperature structures are shown on the left and right sides, respectively. *mF*_o_-*DF*_c_ omit maps of positive (green) and negative (red) peaks at counter levels of indicated sigma value were illustrated using Coot (4).

**Figure S9. Structural deviations between the room and cryogenic temperature structures.** *A*, A plot of distance between Cα atoms against residue number. *B-G*, Superimposition of the room temperature structure (green) and the cryogenic temperature structure (blue). Residues from the symmetry-related molecule are indicated with prime symbols. The two structures were superimposed using LSQKAB (5).

**Supplementary Tables**

**Table S1**

Crystallographic data collection and refinement statistics of the cryogenic temperature structure

| Dataset | WT FoRham1 + Rha (X-ray structure at cryogenic temperature) |
| --- | --- |
| Data collection | X-ray |
| Beamline | PF AR-NE3A |
| Wavelength (Å) | 1.0000 |
| Temperature (K) | 100 |
| Space group | *P*2_1_2_1_2_1_ |
| Unit cell (Å) | *a* = 56.31*, b* = 65.23*, c* = 108.22 |
| Resolution (Å) | 42.63–1.06 (1.08–1.06) |
| Total reflections | 2,252,285 (105,656) |
| Unique reflections | 180,680 (8,886) |
| Completeness (%) | 100.0 (100.0) |
| Redundancy | 12.5 (11.9) |
| Mean I/σ(I) | 12.5 (2.1) |
| *R_merge_ (%)* | 10.3 (107.5) |
| *R_p.i.m_ (%)* | 2.9 (32.3) |
| CC_1/2_^a^ | (0.814) |
| Wilson B-factor (Å^2^) | 8.57 |
| Refinement |  |
| Resolution (Å) | 41.65–1.06 (1.07–1.06) |
| No. of reflections | 180,532 |
| *R*_work_ (%) | 17.2 (25.6) |
| *R*_free_ (%) | 18.3 (26.9) |
| Number of atoms | 3,998 |
| Number of waters | 458 |
| RMSD from ideal values |  |
| Bond lengths (Å) | 0.005 |
| Bond angles (°) | 0.904 |
| Average B-factor (Å ^2^) |  |
| Overall | 15.22 |
| Protein | 13.68 |
| Water | 23.15 |
| Ramachandran plot (%) |  |
| Favored | 96.4 |
| Allowed | 3.6 |
| Outlier | 0 |
| PDB code | 8I4D |

Values in parentheses are for the highest resolution shell.

**Table S2**

Temperature factors of the side chain atoms of His85 and His105 in two states containing HE1 or HE1/DE1

|  | His85 | | His105 | |
| --- | --- | --- | --- | --- |
| Atom name | HE1  State (b) | HE1/DE1  State (c) | HE1  State (b) | HE1/DE1  State (c) |
| CB | 25.13 | 24.85 | 25.72 | 25.49 |
| CG | 25.72 | 25.41 | 25.90 | 25.66 |
| ND1 | 27.32 | 26.94 | 26.50 | 26.20 |
| CD2 | 27.02 | 26.77 | 26.15 | 25.88 |
| CE1 | 28.89 | 28.73 | 26.79 | 26.45 |
| NE2 | 28.60 | 28.31 | 27.20 | 26.88 |
| HB2 | 25.03 | 24.73 | 26.92 | 26.73 |
| HB3 | 27.57 | 27.42 | 25.73 | 25.58 |
| HD2 | 28.96 | 28.59 | 26.97 | 26.84 |
| HE1 | 46.22 (1.00) | 29.30 (0.40) | 37.73 (1.00) | 29.81 (0.73) |
| DE1 | – | 29.07 (0.60) | – | 29.71 (0.27) |

Refined temperature factors are shown in Å^2^. Values in parentheses are refined occupancies.

**Table S3**

Sequences of primers used for mutagenesis

| Name | Sequence^a^ |
| --- | --- |
| D83A (Fw) | 5′-ACGATGGCTGGCCATAACATGATCTCT-3′ |
| D83A (Rv) | 5′-ATGGCCAGCCATCGTCTTCTGAGTATA-3′ |
| D83E (Fw) | 5′-ACGATGGAAGGCCATAACATGATCTCT-3′ |
| D83E (Rv) | 5′-ATGGCCTTCCATCGTCTTCTGAGTATA-3′ |
| D83N (Fw) | 5′-ACGATGAACGGCCATAACATGATCTCT-3′ |
| D83N (Rv) | 5′-ATGGCCGTTCATCGTCTTCTGAGTATA-3′ |
| Y202A (Fw) | 5′-AACGCGGCTATCAACGGACTGGATTAC-3′ |
| Y202A (Rv) | 5′-GTTGATAGCCGCGTTGTTGTCGTCACC-3′ |
| Y202F (Fw) | 5′-AACGCGTTTATCAACGGACTGGATTAC-3′ |
| Y202F (Rv) | 5′-GTTGATAAACGCGTTGTTGTCGTCACC-3′ |
| Y202W (Fw) | 5′-AACGCGTGGATCAACGGACTGGATTAC-3′ |
| Y202W (Rv) | 5′-GTTGATCCACGCGTTGTTGTCGTCACC-3′ |

^a^ Underlined regions indicate the mutated codons.

**Supplementary References**

1. Niimura, N., and Podjarny, A. (2011) *Neutron Protein Crystallography: Hydrogen, Protons, and Hydration in Bio-macromolecules*, Oxford University Press

2. Stanley, P., Moremen, K. W., Lewis, N. E., Taniguchi, N., and Aebi, M. (2022) N-Glycans. in *Essentials of Glycobiology*, 4th Ed (Varki, A., Cummings, R. D., Esko, J. D., Stanley, P., Hart, G. W., Aebi, M., Mohnen, D., Kinoshita, T., Packer, N. H., Prestegard, J. H., Schnaar, R. L., and Seeberger, P. H. eds), Cold Spring Harbor Laboratory Press, Cold Spring Harbor (NY)

3. Neelamegham, S., Aoki-Kinoshita, K., Bolton, E., Frank, M., Lisacek, F., Lütteke, T., O’Boyle, N., Packer, N. H., Stanley, P., Toukach, P., Varki, A., Woods, R. J., Group, T. S. D., Darvill, A., Dell, A., Henrissat, B., Bertozzi, C., Hart, G., Narimatsu, H., Freeze, H., Yamada, I., Paulson, J., Prestegard, J., Marth, J., Vliegenthart, J. F. G., Etzler, M., Aebi, M., Kanehisa, M., Taniguchi, N., Edwards, N., Rudd, P., Seeberger, P., Mazumder, R., Ranzinger, R., Cummings, R., Schnaar, R., Perez, S., Kornfeld, S., Kinoshita, T., York, W., and Knirel, Y. (2019) Updates to the Symbol Nomenclature for Glycans guidelines. *Glycobiology*. **29**, 620–624

4. Emsley, P., Lohkamp, B., Scott, W. G., and Cowtan, K. (2010) Features and development of Coot. *Acta Crystallogr D Biol Crystallogr*. **66**, 486–501

5. Kabsch, W., and IUCr (1976) A solution for the best rotation to relate two sets of vectors. *Acta Crystallographica Section A*. **32**, 922–923
